# Implementation of pre-exposure prophylaxis for human immunodeficiency virus infection: progress and emerging issues in research and policy

**DOI:** 10.7448/IAS.19.7.21108

**Published:** 2016-10-18

**Authors:** Carlos F Cáceres, Annick Borquez, Jeffrey D Klausner, Rachel Baggaley, Chris Beyrer

**Affiliations:** 1 Center for Interdisciplinary Studies in Sexuality, AIDS and Society, Universidad Peruana Cayetano Heredia, Lima, Peru; 2 Division of Global Public Health, University of California, San Diego, CA, USA; 3 David Geffen School of Medicine, University of California, Los Angeles, CA, USA; 4 Fielding School of Public Health, University of California, Los Angeles, CA, USA; 5 HIV Department, World Health Organization, Geneva, Switzerland; 6 Center for Public Health and Human Rights, Bloomberg School of Public Health, Johns Hopkins University, Baltimore, MA, USA

**Keywords:** HIV, pre-exposure prophylaxis, implementation science, combination prevention, health systems, acceptability, program cost, scale-up

## Abstract

**Background:**

In this article, we present recent evidence from studies focused on the implementation, effectiveness and cost-effectiveness of pre-exposure prophylaxis (PrEP) for HIV infection; discuss PrEP scale-up to date, including the observed levels of access and policy development; and elaborate on key emerging policy and research issues to consider for further scale-up, with a special focus on lower-middle income countries.

**Discussion:**

The 2015 WHO Early Release Guidelines for HIV Treatment and Prevention reflect both scientific evidence and new policy perspectives. Those guidelines present a timely challenge to health systems for the scaling up of not only treatment for every person living with HIV infection but also the offer of PrEP to those at substantial risk. Delivery and uptake of both universal antiretroviral therapy (ART) and PrEP will require nation-wide commitment and could reinvigorate health systems to develop more comprehensive “combination prevention” programmes and support wider testing linked to both treatments and other prevention options for populations at highest risk who are currently not accessing services. Various gaps in current health systems will need to be addressed to achieve strategic scale-up of PrEP, including developing prioritization strategies, strengthening drug regulations, determining cost and funding sources, training health providers, supporting user adherence and creating demand.

**Conclusions:**

The initial steps in the scale-up of PrEP globally suggest feasibility, acceptability and likely impact. However, to prevent setbacks in less well-resourced settings, countries will need to anticipate and address challenges such as operational and health systems barriers, drug cost and regulatory policies, health providers’ openness to prescribing PrEP to populations at substantial risk, demand and legal and human rights issues. Emerging problems will require creative solutions and will continue to illustrate the complexity of PrEP implementation.

## Introduction

The timely publication, in September 2015, of the World Health Organization (WHO) Early Release Guidelines on Use of Antiretrovirals for HIV Treatment and Prevention [1] not only confirmed the recommendation of oral HIV pre-exposure prophylaxis (PrEP) as an additional prevention strategy for members of key populations but also extended it to any person at substantial risk. This has reinvigorated discussions on combination prevention, by adding a new, effective prevention choice, and requires efforts to facilitate scale-up where it might be most beneficial.

Globally in mid-2016, PrEP scale-up is just starting. Many countries are beginning to consider if and how PrEP could be employed in their HIV responses to increase impact. To date, roughly 50,000 people are on PrEP in the United States, the country with the greatest experience in PrEP delivery. The U.S. Centers for Disease Control suggest 1.2 million persons at substantial HIV risk, particularly men who have sex with men (MSM), should be on PrEP in the United States [2].

Recently, a number of countries in Africa, Asia, Europe and Latin America have started measures to enable PrEP implementation, such as the approval of tenofovir–emtricitabine for prevention; the inclusion of PrEP in the national prevention policies and guidelines; and the conduct of implementation research or other efforts to define roll-out conditions (see below). Nevertheless, most countries have not taken any steps yet, due to concerns about local relevance, costs and sustainable funding [3], potential competition with treatment expenditures and other health systems issues (e.g. prioritizing potential beneficiary populations, implementation concerns about safety, drug resistance, adherence and “risk compensation”). Stigma and discrimination against some key populations often result in poor access to health services in general and inadequate prioritization of their needs, contributing to low PrEP awareness among some key population groups. Conversely, some communities of MSM, much more aware of PrEP, are establishing informal channels to obtain PrEP medications in locations where they are officially unavailable and lobbying for PrEP availability.

Building from our 2015 commentary [4], in this article we will present evidence of the implementation process of PrEP programmes in 2015–2016, with data on potential impact and cost-effectiveness when available; discuss correlates of the observed levels of access and policy development; and analyze key emerging policy and research issues to consider for further scale-up, with a special focus on lower-middle income countries (LMIC).

## Discussion

### Can PrEP curb HIV incidence?

A crucial factor in the relevance of PrEP scale-up in a combination prevention framework is its estimated impact on HIV incidence and cost-effectiveness. A few recent modelling studies, and reviews of such studies, indicate that consideration of the local epidemic context, including the current coverage of other interventions, is key to achieving efficiency:

A systematic review focused on seven modelling studies published in 2015 that evaluated the cost-effectiveness of introducing PrEP. Three of these were in LMIC and investigated the introduction of PrEP among African heterosexual serodiscordant couples. The incremental cost-effectiveness ratio varied between $5000 and $10,000 per disability-adjusted life year (DALY) averted, when PrEP was used for a limited time period before and after the HIV-positive partner had initiated antiretroviral therapy (ART) [5]. In South Africa, such an intervention was found to be cost-effective under a threshold of three times the national GDP/capita. In Nigeria, it was found to be cost-effective under this threshold only if PrEP was integrated in addition to condom promotion and treatment as prevention. In Uganda, PrEP in addition to ART was not found to be cost-effective under this threshold. In all studies, PrEP cost-effectiveness was improved when “infections averted” was used as the outcome (as opposed to DALYs averted), or when taking a longer-term perspective. This is due to PrEP being a prevention intervention, and as such, its impact on HIV-associated DALYs is not apparent on a 10-year or even 20-year time frame.

Another modelling study [6] investigating the introduction of PrEP among women in Western Kenya found that it could have a substantial impact on incidence (22–28% reduction over four years) if implemented alone and that in combination with increased voluntary medical male circumcision and the implementation of 2013 WHO ART guidelines it could reduce incidence by 46–67%, but cost-effectiveness was not evaluated. Cremin and Hallet [7] used Nyanza, Kenya, as a case study to investigate PrEP intervention efficiencies associated with longer residual protection and changes in cost, adherence and prioritization as a function of coverage and time. They found that the ability to adequately prioritize PrEP to groups with high HIV incidence was the strongest determinant of intervention efficiency and highlighted the extent to which dynamic interactions could affect PrEP intervention impact, warning policy makers of the importance of considering the potential effects of programme scale and duration on efficiency.

Mukandavire *et al*. [8] investigated the impact of PrEP among female sex workers (FSW) in comparison with that of increasing condom use and found that the latter was likely to be larger given its greater efficacy and its effect on both acquisition and transmission, resulting in the protection of both FSW and their clients. When including non-commercial partners of FSW, the relative impact of PrEP over condoms improved but only substantially when the contribution of these partners to HIV transmission was assumed to be high. The authors conclude that PrEP could be a valuable prevention tool among FSW once condom interventions have been maximized and to protect FSW who are unable to increase their condom use. In a study where PrEP introduction among transgender women (TW) sex workers in Peru was considered along with other four strategies, all possible combinations were modelled for joint effectiveness and cost-effectiveness. At current retail Truvada^®^ prices, it was found that the inclusion of PrEP was effective but required lower costs to become cost-effective [9].

The focus of some modelling studies on populations at high risk of infection over limited periods of time and in combination with other interventions, compared to that of earlier modelling studies that explored large PrEP scale-up, suggests the recognition of the need for a strategic implementation of PrEP to increase its cost-effectiveness. In addition, as pointed by Cambiano *et al*. [5], cost-effectiveness does not necessarily translate into affordability, and therefore, budget impact analyses that carefully consider implementation and funding strategies are the necessary next step to plan for PrEP scale-up.

As of mid-2016, empirical evidence of effectiveness is not yet available from LMIC. In the United States, referrals for and initiation of PrEP increased in clinical practice since 2012, with very low rates of HIV acquisition among adherent PrEP users. However in one study, high rates of sexually transmitted infections (STIs) continue to be reported, as were seen in the delayed arm in PROUD, and reported decreases in condom use were seen in a small subset of PrEP users [2]. Similarly, a three-city demonstration project in the United States found that annualized HIV incidence was very low despite high incidence of other STIs [10], and the pivotal PROUD study among MSM in sexual health clinics in the UK found an effectiveness greater than efficacy in iPrEX, in part due to reaching high-risk individuals who were motivated to take PrEP and whose adherence was high. HIV incidence was very low but bacterial STI incidence has been high, indicating that high-risk MSM are using PrEP and that STI screening is an important part of PrEP delivery [11].

### Global PrEP scale-up, as of early 2016

Internationally, HIV prevention implementers and funders are beginning to recognize the potential of PrEP and support implementation efforts (including formative research, demonstration and pilot projects) in lower-middle income countries. By the end of 2016, WHO will be releasing full PrEP implementation guidelines to support countries to provide PrEP safely and effectively to a range of populations and in various settings. UNAIDS is recognizing PrEP for populations at substantial risk as an important intervention and has included PrEP in the UN global Fast Track prevention targets [12]. The Global Fund to fight AIDS, tuberculosis and malaria is willing to fund PrEP components of combination prevention in eligible countries according to their overall plans and regulations for types of countries [13]. The U.S. President's Emergency Plan for AIDS Relief (PEPFAR) new policy is supporting the provision of PrEP to young women and sex workers in Africa through the DREAMS initiative, as well as to people in serodiscordant couples (as a bridge to antiretroviral therapy in positive partners) and to people at substantial risk in key populations of MSM, female sex workers and injection drug users [14]. UNITAID is considering funding PrEP implementation science projects in LMIC regions [15].

As indicated, the number of countries where any PrEP-related policy has already been put in place is limited (see Table 1). Only three countries – the United States and France and, recently, South Africa, have formally incorporated PrEP in their regular HIV prevention programming. In June 2016, South Africa started to provide PrEP for sex workers as a national programme, and other countries in the region are planning for PrEP and developing national policies and guidelines for implementation. We observe two other kinds of activities: regulatory changes (approval of the use of tenofovir–emtricitabine for PrEP) and an increasing number of ongoing or planned projects and programmes to deliver PrEP.

**Table 1:** Countries adopting policy changes leading to potential HIV pre-exposure prophylaxis (PrEP) scale-up

Country	TDF-containing drug approved for PrEP	Demonstration projects	National guidelines for PrEP	Source of funding
Australia	Effective	Ongoing	Effective	Domestic
Brazil	Pending	Ongoing (1); Pending approval (2)	Planned (3)	(1) US NIH; (2) UNITAID (3) Domestic
Canada	Effective			
India	Generic can be sold for prevention	Planned/ongoing	–	BMGF
France	Effective		Effective (daily and on-demand use)	Domestic
Kenya	Effective	Planned/ongoing	Effective	BMGF and others
Malaysia	Generic can be sold for prevention		Effective for out-of-pocket use	Out of pocket
Mexico	–	Pending approval	–	UNITAID
Mozambique	–	Planned/ongoing		BMGF and others
Peru	Effective	Ongoing (1); Pending approval (2)		(1) amfAR (2) UNITAID
Philippines	–	Planned/ongoing		Several sources
South Africa	Effective	Planned/ongoing	Effective	Several (domestic and foreign)
Thailand	Generic can be sold for prevention	Ongoing/planned/pending approval	Effective for out-of-pocket	Several (domestic and foreign)
United States	Effective	Completed/ongoing	Effective	Domestic
United Kingdom	–	Completed/ongoing	Pending	Domestic
Zimbabwe	–	Ongoing/pending approval		Several sources

BMGF, Bill and Melinda Gates Foundation; PrEP, pre-exposure prophylaxis.

### Awareness/acceptability of PrEP among stakeholders

As the number of recent studies on PrEP has grown over the past 18 months, here we are focusing on publications from LMIC, as those face greater challenges for implementation.

#### Healthcare providers

Data from LMIC are limited. In a qualitative study in Peru (2014–2015), providers maintain limited awareness about PrEP and express skepticism about its use in prevention, linked to drug complexity, cost and presumed risk compensation [16].

#### General public

A study to explore the public opinion, community interest and perceptions about the use and access to PrEP in Nigeria concluded that increasing PrEP uptake by HIV serodiscordant couples requires motivating the HIV-negative male partners and establishing effective stigma reduction strategies [17].

#### Potential and current users

PrEP use has been associated with feelings of empowerment, agency and safety during sex, and partners’ honesty in sharing their HIV status [18]. Data from LMIC are limited. In Peru in 2014, among MSM communities and stakeholders alike, prior to the licensing of Truvada^®^ for PrEP, condoms were still described as the mainstay of prevention, yet condom use among MSM has always been insufficient. The men interviewed for the study showed some knowledge of PrEP, coexisting with skepticism about potential utilization [11].

One study among MSM and female sex workers participating in a PrEP trial in Kenya in 2009–2010 reported that PrEP acceptability was high. Adherence was 83% (interquartile range [IQR] 63–92) for daily PrEP, 55% (IQR 28–78) for fixed intermittent dosing and 28% (IQR 14–50) for post-coital dosing. Social impacts (stigma, rumours and relationship difficulties due to being perceived as HIV-positive) were prevalent. Qualitative data on adherence barriers and better measurements of sexual activity are necessary to determine whether adherence to post-coital PrEP is comparable to more standard regimens [19,20]. HPTN 067/ADAPT's three randomized trials, assessing the feasibility and acceptability of different PrEP regimens with three different populations, in Bangkok, Harlem (New York) and Cape Town found, across all sites, that adherence was higher for the daily rather than the non-daily doses. For example, in Bangkok, 85% of daily doses, 79% of twice-weekly doses and 65% of event-driven doses were taken as prescribed. In Harlem, the respective figures were 65, 46 and 41%. Several advantages of daily regimens were identified – likely to offer more protection, more forgiving of missed doses and helping people develop habits of daily pill-taking [21].

A high uptake of PrEP, and retention in PrEP for the duration of risk, was found among serodiscordant couples in the Partners Demonstration Project, an open-label implementation study evaluating integrated delivery of PrEP and ART in Kenya and Uganda [22]. A sub-study used qualitative methods to gather insights into couples’ early experiences with PrEP use. Couples reported that PrEP offered them an additional strategy to reduce HIV risk, meet their fertility desires and cope with HIV serodiscordance; remaining HIV negative at follow-up visits, as well as their providers’ advice and service friendliness, reinforced couples’ decisions and motivated continued adherence to PrEP [23].

### Health systems: challenges and strategies

Health systems are at the core of PrEP implementation. Remaining and emerging challenges should be addressed with policy dialogue and implementation research.

#### Who pays for PrEP?

As potential PrEP users often report inconsistent condom use, in the long term paying for PrEP during people's higher-risk life periods is more affordable than paying for lifelong ART [24]. Nevertheless, after the publication of the WHO recommendations, sustainable funding has become a key issue to consider in both high-income countries and LMIC before deciding to establish PrEP programmes. In the UK, which was home to the successful PROUD study [11], the National Health Service has not as yet been able to fund PrEP [25], given that local authorities are the responsible commissioner for HIV prevention services, leading to community mobilization to demand access [3].

In high-income countries and in upper-middle income signatories of free-trade agreements, tenofovir-emtricitabine (TDF-FTC) is only available as very costly Truvada^®^ (although the expiration of its patent protection in 2017 may change this). Conversely, generic TDF-FTC is already being produced and is available for less than USD10 per month, for the benefit of LMIC with a high burden of HIV infection [26]. Some United Nations partners have established regional procurement mechanisms to increase drug access, too.

#### Selecting the right drug

Clinical trials such as Partners’ PrEP showed comparable efficacy between the TDF-FTC and tenofovir-only arms [27]; likewise, the Bangkok PrEP study also found efficacy for a TDF-based PrEP regime for people who inject drugs [28]. Given the partial availability of TDF-FTC in the world at present, the WHO recommendations defined oral PrEP as the use of tenofovir-containing pills. Although high efficacy for TDF alone was found for the prevention of heterosexual HIV transmission, its comparative effectiveness in preventing HIV transmission in anal sex between men is not proven. In addition, given the treatment equivalence of lamuvidine (3TC) and FTC, and higher availability of such drug in some countries, WHO guidance is forthcoming on 3TC as an alternative to FTC in combination with TDF for PrEP.

#### Ensuring safety

Based on the evidence available, the WHO Early Release Recommendations [1] for HIV Treatment and Prevention in both higher- and LMIC have indicated that before starting PrEP, subjects’ baseline renal function should be assessed, together with HIV status [1]. Safety monitoring data from PrEP programmes in LMIC are still not available. To assess the relative importance of the adverse effects of TDF-FTC used for PrEP, a recent study compared the number needed to harm (NNH) for TDF-FTC in MSM, transwomen and seronegative partners in heterosexual serodiscordant couples, with adverse effects of aspirin used for the prevention of cardiovascular disease among middle-aged men and women [29]. It was found that, if the populations and the frequency of use are comparable, the NNH for TDF-FTC compared favourably with the NNH for aspirin concerning adverse effects.

#### Defining delivery models with appropriate monitoring and optimal adherence

The administration of PrEP implies coordination of various components: initial eligibility assessment (including HIV testing), regular follow-up testing, safety and adherence monitoring, and drug supply. HIV testing is fundamental to confirm eligibility and rule out subsequent infection. As HIV testing and counselling in LMIC is often facility-based, to increase testing of people at substantial HIV risk who do not visit clinical services, other approaches such as workplace, outreach, mobile and potentially HIV self-testing should be considered [30,31]. Links with community-based organizations and key population networks to support linkage following testing are essential to ensure people living with HIV link to ART services and that those HIV-negative link to prevention services where PrEP and other options are available.

People taking PrEP require regular follow-up, at least quarterly for HIV and STI testing and periodic kidney function assessment. There are many different possible approaches for PrEP delivery and possibilities for integrating into other clinical services – for example STI, reproductive health services or other services for key populations. Task-sharing should be considered for PrEP delivery with nurse and clinical officer-led services. A PrEP demonstration project conducted in three different settings in San Francisco recently reported that the integration of PrEP into busy clinical settings is feasible [32].

Integration of PrEP with relevant services will increase sustainability and foster comprehensive care. Recent programmatic data from the United States indicate that as PrEP programmes must deal with substantial rates of other STI, they provide an opportunity to integrate periodic STI screening and management [33].

The various barriers to reaching and delivering services for adolescent girls and young women in settings of high incidence in southern and East Africa [34–37] should be identified and tackled [38]. Demonstration projects to explore PrEP implementation in those populations are a priority. Medication adherence in younger women has also been recognized as a significant challenge [39,40]. Risk assessment tools have been designed for men who have sex with men (MSM) and people who inject drugs (PWID) but have not been critically evaluated yet in terms of their usefulness in PrEP implementation. Regardless of population and point of entry, evidence-based strategies to support adherence should be considered, without losing a user-centred approach, where risk reduction is the goal – rather than PrEP adherence per se [41]. Within the Partners PrEP study among HIV-negative members of serodiscordant couples in Kenya and Uganda, objective measures of PrEP adherence were collected using unannounced home-based pill counts and electronic pill bottle monitoring; participants received individual and couples-based adherence counselling at PrEP initiation and throughout the study; participants were followed monthly and counselling was intensified if unannounced pill count adherence fell to <80%. Median adherence was 99.1% (IQR 96.9–100%) by unannounced pill counts and 97.2% (90.6–100%) by electronic monitoring over 807 person-years [42].

#### Improving provider training

Given the need to prioritize individuals at high risk of infection to improve PrEP cost-effectiveness, training of health providers to better identify potential PrEP beneficiaries will be important. Sexual history training among a sample of U.S. physicians resulted in increased frequency of documented sexual history discussions and greater comfort with sexual health discussions [43]. Risk assessment tools relying on self-reported risk behaviours to estimate HIV risk have been successfully implemented among MSM [44] and PWID [45]. Applying algorithms to routinely collect behavioural or STI history data from patients’ electronic health records provides additional support in this task [46].

### Involving communities and dealing with structural barriers

Meaningful, effective community involvement is the other key factor in PrEP scale-up [47]. The prevailing legal and human rights barriers to access care affecting MSM, TW and sex workers may pose significant challenges to providing PrEP to these key populations, in many settings, particularly in LMIC in Africa and the Caribbean where homosexuality and sex work remain criminalized [48,49]. PrEP scale-up for key populations provides an opportunity to improve services and increase service utilization among key populations as well as possibility of “programmatic risk” [50].

### Using implementation research

Focused implementation research can be used to resolve some of the challenges described above, such as dynamic stakeholders’ attitudes and information needs; PrEP awareness and demand creation; best options for the target drug (i.e. source, purchase mechanism and funding); refinement of target population(s); programme organization, modes of delivery and integration with other services; health provider training; and strategies to manage STIs and tackle structural barriers to programmatic success [51].

## Conclusions

The 2015 WHO recommendation to support the offer of PrEP to people at substantial HIV risk was based on compelling effectiveness evidence to prevent HIV acquisition in various populations. To date, PrEP implementation outside the United States seems to be moving to a new phase, given increasing numbers of demonstration and pilot programmes underway or being considered. The challenge is to learn from those and support wider use for populations that continue to experience high incidence and for whom additional prevention choices are needed. Community advocates and networks in many countries are leading the demand for greater access. Many challenges remain for implementation in LMIC, but the evidence of effectiveness of PrEP, the acceptability and uptake by people who could benefit most and the cost-effectiveness of PrEP if offered to people at highest risk are compelling reasons to push for greater availability and implementation. It is hoped that future long-acting formulations such as injectable [52] will be effective, and the experience gained from implementing daily PrEP will be crucial to facilitate their introduction.
